# Supplementary figures and images for: Prophage-encoded Hm-oscar gene recapitulates Wolbachia-induced male-killing in the tea tortrix moth Homona magnanima
Source: eLife. 2025 Apr 14;13:RP101101. doi: 10.7554/eLife.101101 (PMC11996169; doi:10.7554/eLife.101101)

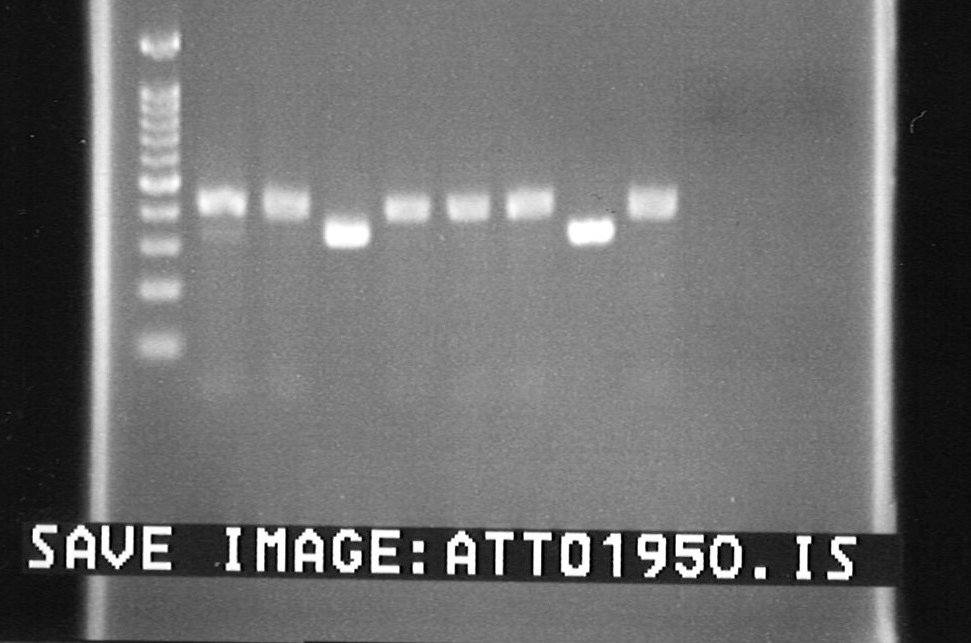

Supplement: Figure 2—source data 2. [file elife-101101-fig2-data2.zip › figure2-source data2/ATTO1950.png]
