# Supplementary material for: Prophage-encoded Hm-oscar gene recapitulates Wolbachia-induced male-killing in the tea tortrix moth Homona magnanima
Source: eLife. 2025 Apr 14;13:RP101101. doi: 10.7554/eLife.101101 (PMC11996169; doi:10.7554/eLife.101101)
Supplement: Figure 2—source data 3. [file elife-101101-fig2-data3.pdf]

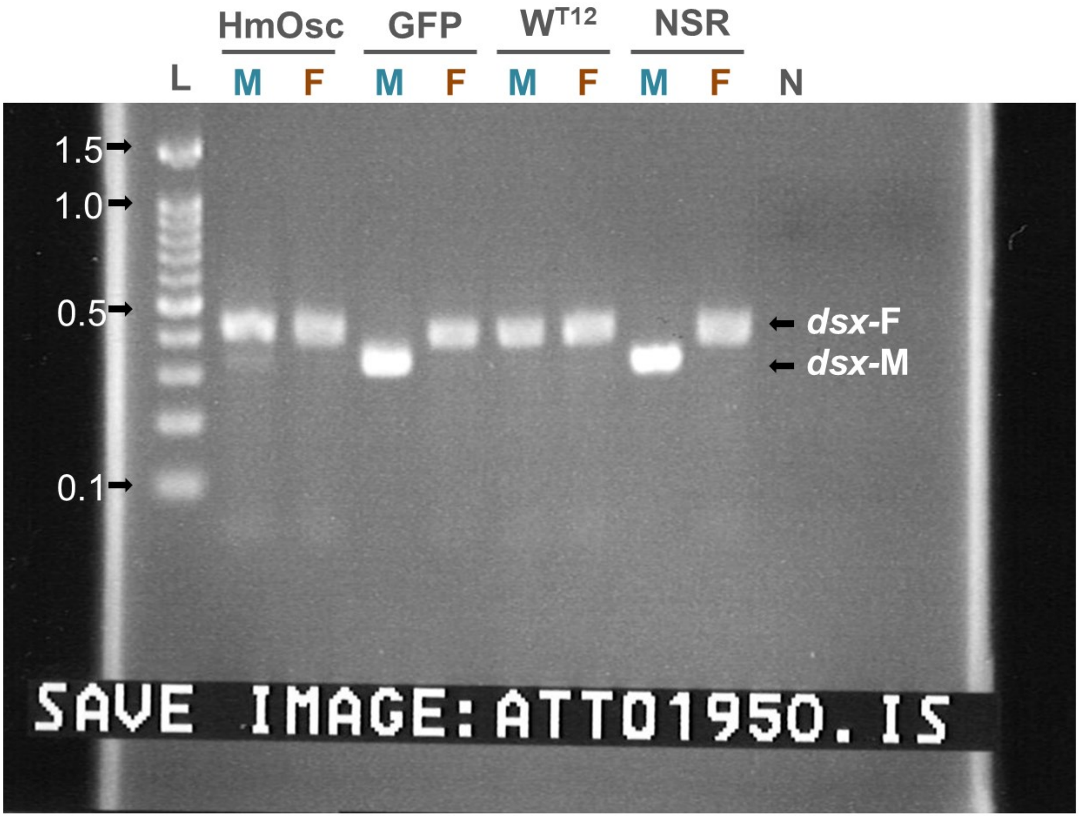

Figure 3, Source Data 3. Splicing patterns of the downstream sex-determining gene *dsx* of *H. magnanima* embryos (5 days post oviposition). Abbreviations: HmOsc, *Hm-oscar* injected group; GFP, GFP-injected group; W^{T12}, *w*Hm-t-infected line; NSR, non-infected/injected line. M and F indicate W chromatin-negative (ZZ: male genotype) and W chromatin-positive (ZW: female genotype) mature embryos, respectively. *dsx-F* and *dsx-M* represent female and male-specific splicing variants, respectively. L: 100 bp DNA ladder (ExcelBand 100 bp DNA Ladder, SMOBIO Technology, Inc., Hsinchu, Taiwan). 0.1, 0.5, 1.0, and 1.5 kb markers are indicated with arrows. N: negative control (water).
